# Evolution and function of interleukin-4 receptor signaling in adaptive immunity and neutrophils

**DOI:** 10.1038/s41435-020-0095-7

**Published:** 2020-03-06

**Authors:** Lukas E. M. Heeb, Cecilie Egholm, Onur Boyman

**Affiliations:** 1 Department of Immunology, University Hospital Zurich, CH-8091 Zurich, Switzerland; 2 Faculty of Medicine, University of Zurich, CH-8006 Zurich, Switzerland

**Keywords:** Interleukins, Neutrophils, Adaptive immunity

## Abstract

The cytokines interleukin (IL)-4 and IL-13, signaling via the IL-4 receptor (IL-4R), orchestrate type 2 immunity to helminth infections and toxins. Activation of epithelial and myeloid cells, and a transient neutrophils influx initiates type 2 immune responses, which are dominated by basophils, eosinophils, mast cells, B cell immunoglobulin E production, and type 2 T helper and T follicular helper cells. Interestingly, IL-4 and IL-13 can curtail chemotaxis and several effector functions of neutrophils in mice and humans. This inhibitory role of IL-4 and IL-13 probably developed to limit tissue damage by neutrophils during type 2 immunity where a “weep and sweep” response aims at expulsion and decreased fecundity, instead of killing, of macroparasites. Here, we review when IL-4R signaling cytokines appeared during evolution relative to neutrophils and adaptive immunity. Neutrophil-like granular phagocytes were present in invertebrates throughout the bilaterian clade, but we were unable to find data on IL-4, IL-13, or their receptors in invertebrates. Conversely, vertebrates had both adaptive immunity and IL-4, IL-13, and IL-4Rs, suggesting that type 2 cytokines evolved together with adaptive immunity. Further studies are necessary to determine whether IL-4R signaling in neutrophils was established simultaneously with the appearance of adaptive immunity or later.

## Introduction

Interleukin (IL)-4 and IL-13 are well known for their key roles in type 2 immune responses, which result in resistance to helminth parasites and inactivation of toxins. IL-4 and IL-13 induce differentiation of naïve T cells to type 2 T helper and T follicular helper cells, B cell antibody production and isotype switching to immunoglobulin E (IgE), expansion of basophils and eosinophils, mast cell activation, skewing of macrophages toward the subtype of alternatively-activated macrophages (also known as type 2 or M2 macrophages), and goblet cell hyperplasia [1, 2].

It is well established that neutrophils are present in type 1 and type 3 immune responses, which serve to fight intracellular and extracellular pathogens, respectively. However, recent evidence has revealed a role for neutrophils in protection against parasite infections [3, 4]. Thus, it was shown that during the initial phase of type 2 responses, the presence of neutrophils was beneficial for limiting parasite survival and spreading. This was primarily due to formation of neutrophil extracellular traps (NETs) and degranulation [5]. Accordingly, also in type 2 immune responses, neutrophils seem to be the first nonresident immune cells to arrive to the affected site. Despite their very short lifespan, neutrophils are able to shape the immune response long after their death, for example by guiding and attracting other immune cells or by their ability to prime macrophages to become M2 macrophages [6]. These M2 macrophages are efficient in protecting during a secondary infection. Thus, neutrophils do not only leave a temporary mark but are able to impact future immune responses.

However, there is accumulating evidence showing that IL-4- and IL-13-mediated IL-4 receptor (IL-4R) signaling in both mouse and human neutrophils inhibits their migration and effector functions in vitro and in vivo [7, 8]. In a number of different mouse models including sterile inflammation, bacterial infection, helminth infestation, and rheumatoid arthritis, IL-4R signaling was shown to have an inhibitory effect on neutrophils [9–12]. Human neutrophils isolated from allergic patients, a condition dominated by the presence of IL-4 and IL-13, were less capable of migrating and producing NETs than neutrophils from healthy donors [13]. Thus, we hypothesize that inhibition of neutrophil effector functions in type 2 immune responses constitutes a crucial effect of the IL-4/IL-13–IL-4R system. Failure of this regulatory system can cause detrimental tissue damage, as seen with neutrophilic types of asthma.

Why neutrophils are beneficial for type 2 immune responses and, simultaneously, type 2 cytokines restrict neutrophil effector functions, can be explained when considering the timing of events. During the initiation phase of a type 2 immune response, there is little or no type 2 cytokines present, and neutrophils are needed as a first wave of defense. Once the type 2 immune response is fully active, abundant IL-4 and IL-13 suppress neutrophil effector functions, which at this stage—via neutrophil degranulation and NET formation—would cause excessive tissue damage. Thus, timed IL-4R signaling in neutrophils allows early influx but limits tissue damage by neutrophils during the “weep and sweep” phase of type 2 immunity.

Considering this IL-4R-mediated mechanism of neutrophil regulation, we wondered whether IL-4R signaling cytokines initially evolved to refine adaptive immune responses against parasites or to provide timed inhibition of innate immune cells, such as neutrophils, to limit tissue damage. In order to address this question, we reviewed and combined phylogenetic data on neutrophils, the adaptive immune system, and the IL-4/IL-13–IL-4R system.

## The evolution of neutrophils

Neutrophils are the most abundant leukocytes in human blood and are typically the first nonresident immune cells to respond to an inflammatory or infectious stimulus [14]. Thus, together with barrier epithelial cells and resident immune cells, neutrophils form the first line of defense to limit pathogens until the adaptive immune response arrives [15]. Neutrophils are able to fight infection by phagocytosis, release of antimicrobial effector molecules (termed degranulation), production of reactive oxygen species (ROS), and the formation of NETs, which are DNA meshes decorated with antimicrobial peptides that neutrophils can expulse in response to pathogens that are too large to phagocytose [8, 16–19].

**Fig. 1:**
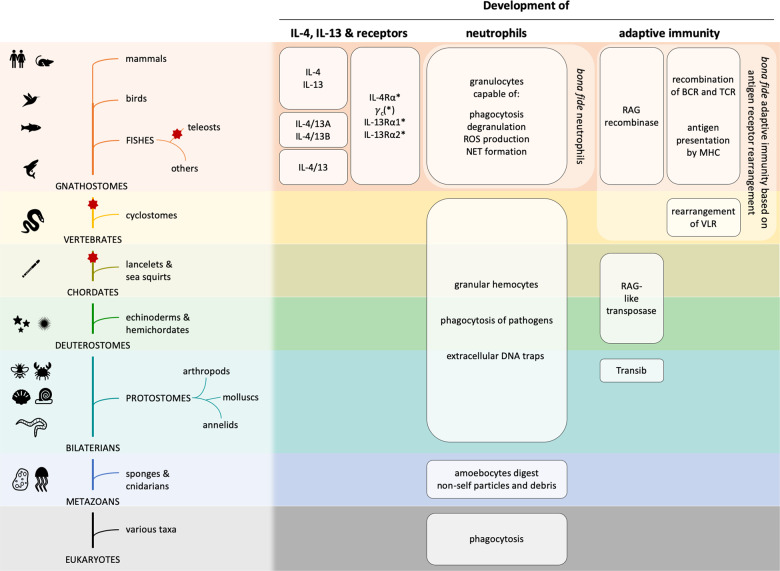
Evolutionary timelines of IL-4, IL-13 and their receptors, neutrophils, and adaptive immunity. Left: Phylogenetic tree showing the taxa mentioned in this review, to be read from bottom to top. Eukaryotes encompass a variety of different taxa, one of them being metazoans (i.e., animals). Bilaterians (i.e., animals displaying bilateral symmetry, at least at embryonic stages) are a major subgroup of metazoans and separated from sponges and cnidarians (e.g., jellyfishes, corals). Bilaterians can be divided into protostomes and deuterostomes, the former of which include arthropods (e.g., insects, crustaceans, spiders), molluscs (e.g., snails, clams), and annelids (e.g., earth worms, leeches). Deuterostomes encompass chordates as well as echinoderms (e.g., starfishes, sea urchins) and hemichordates (e.g., acorn worms). The vertebrates are a major clade within the chordates. Examples of invertebrate chordates are lancelets and sea squirts. The vertebrates in turn are further divided into gnathostomes (i.e., jawed vertebrates) and jawless vertebrates, of which cyclostomes (e.g., lampreys, hagfishes) are the only extant members. The main clades of the gnathostomes discussed in this review are fishes, birds, and mammals. Here, we also indicate the branching of teleost fishes. Color changes indicate the entry into a new clade (in capitals) on the way to mammals, becoming increasingly narrow toward the top. Icons on the left side illustrate representative species from the taxa on the same level (e.g., honeybee - arthropods, sea urchin - echinoderms, earth worm - annelids). Red stars mark whole genome duplication events. Right: Occurrences of different aspects of neutrophil biology, type 2 cytokines, and adaptive immunity in evolutionary context. Depicted features are not necessarily present in the entire clade or taxon (e.g., Transib has so far been identified in some insect species, but not in other arthropods such as crustaceans.). Although genes related to the vertebrate RAG have been found in invertebrates, the recombinase function of modern-day RAG is only present in vertebrates, while RAG-like genes in invertebrates are either absent, silent, encode transposase enzymes, or their function is unknown. Asterisks (*) indicate the presence of two receptor variants in teleost fishes owing to a third round of whole genome duplication. For *γ*_c_, however, the presence of two variants might not be solely due to whole genome duplication events. BCR B cell receptor, *γ*_c_ common γ chain cytokine receptor, IL-4 interleukin-4, IL*-*4R interleukin-4 receptor, MHC major histocompatibility complex, NET neutrophil extracellular trap, RAG recombination-activating gene, ROS reactive oxygen species, TCR T-cell receptor, VLR variable lymphocyte receptor.

Phagocytosis, one of the key effector functions of mammalian neutrophils, is a ubiquitously present process throughout nature from unicellular amoebae to multicellular organisms [20]. In basic invertebrates, such as sponges or cnidarians, specialized phagocytic cells called amoebocytes are responsible for taking up foreign material and debris, but in some cases also food particles [21–23]. Protostomes and invertebrate deuterostomes all have more or less complex innate immune systems consisting of non-granular and granular hemocytes. Hemocytes are mesoderm-derived cells that recognize and phagocytose nonself particles and release antimicrobial granules, thus being reminiscent of monocytes, macrophages, and granulocytes of higher vertebrates [23, 24]. The demonstration of DNA extracellular trap formation not only in mammalian neutrophils and eosinophils [25], but also in granulocytes of fish [26], crustaceans [27], molluscs [28, 29], and worms [30], provides further evidence of functional analogies between mammalian and invertebrate granulocytes. Also the production of ROS by oxidase enzyme complexes has been shown in numerous invertebrate species [31]. Moreover, histological stainings of invertebrate granular hemocytes show acidophilic (i.e., eosinophilic), basophilic, and neutrophilic cells with multi-lobulated nuclei [23]. All these striking morphological and functional parallels lead to the conclusion that granular phagocytes (i.e., neutrophils) are a well-conserved and phylogenetically ancient immune cell type (Fig. 1).

## The evolution of adaptive immunity

The adaptive immune system of jawed vertebrates (gnathostomes) centers around the genes responsible for recombination of antigen receptors. The evolution of this branch of immunity is closely linked to two major evolutionary events: the emergence of recombinase-activating gene 1 and 2 (RAG1 and RAG2) and the occurrence of several rounds of whole genome duplication (WGD).

In gnathostomes, RAG proteins are expressed in developmental stages of B and T cells, and are responsible for the random joining of one variable, one joining, and—in some cases—one diversity gene segment of the antigen receptor gene locus. This process, also termed V(D)J recombination, allows the creation of a vast variety of different receptors from a relatively low number of single gene segments [32]. *Rag* or *Rag*-like genes can be found throughout the superphylum of deuterostomes, and a gene related to *Rag1* called *Transib* was also found in insects (e.g., *Helicoverpa zea*). Surprisingly, Transib and RAG1 proteins have very similar enzymatic activity and specificity and the catalytic triad is conserved in both [33]. This suggests that the ancestor of modern-day *Rag* was acquired by a common ancestor of protostomes and deuterostomes. While *Rag*-like genes are ancient and well conserved, their function changed during evolution: *Transib* and active *Rag*-like loci in invertebrates act as transposons, i.e., DNA segments coding for a protein that excises their own DNA segment and inserts it at another site in the genome. RAG proteins in jawed vertebrates, however, act as recombinases; they do not excise their own gene but DNA in between variable, diversity, and joining gene segments [34]. Interestingly, cyclostomes, the only living jawless vertebrates, do not have *Rag* but they have an adaptive immune system based on recombination of leucine-rich repeats leading to the generation of specific agglutinins called variable lymphocyte receptors that are membrane-bound or secreted [32]. Collectively, the presence of *Rag*-related genes is widespread throughout the bilaterian clade, but only gnathostomes use RAG as a recombinase which enables the development of a *bona fide* adaptive immune system (Fig. 1).

It is now widely accepted that the genome of a common vertebrate ancestor underwent two rounds of WGD, resulting in a fourfold amount of DNA [35]. This increase in accessible raw material made it possible to refine and diversify the genome. Refinement can be achieved by subfunctionalization, a process by which the functionalities of the original gene are distributed among its daughters, which can then evolve to become specialized genes [36]. By having multiple copies of the same gene, one of them can be freed from selective pressure and can accumulate mutations, potentially resulting in new genes with new functions in a process called neofunctionalization, hence diversifying the genome [36]. An immunologically relevant example is the quadruplication of the proto-major histocompatibility complex (MHC) chromosome that gave rise to four paralogous regions all coding for genes involved in antigen presentation and recognition [37, 38].

In conclusion, the founding stones for the establishment of an adaptive immune system existed already in primitive bilaterian ancestors, but an enzyme capable of recombination rather than translocation only occurred in jawed vertebrates. Thus, *bona fide* adaptive immunity is solely present in gnathostomes and must have appeared first in a common ancestor.

## The evolution of IL-4 and IL-13

Genes encoding for proteins related to signaling in the immune system are under a constant evolutionary pressure to adapt and shape immunity toward the most favorable protection of the host. This is nicely illustrated by the finding that among the top 25 genes showing the highest degree of evolutionary divergence between mouse and human orthologues, 7 encode for cytokines or cytokine receptors [39]. Due to their low homology even within mammals, the genes encoding for IL-4 and IL-13 are difficult to track in other species. In the mammalian genome, *Il4* and *Il13* are placed side by side and researchers are therefore often searching for both *Il4*- and *Il13*-linked genes as well as flanking genes such as *Kif3a* and *Rad50* that are much better conserved [40]. With the increasing number of genomes being sequenced, a substantial amount of evidence is emerging to shed light on the evolution of these and other genes. *Il4*/*Il13*-related genes have been found in a number of both fish and bird species and even in amphibians although the latter has not been confirmed by functional studies (Fig. 1) [40–42]. A single *Il4*/*Il13*-related gene was found in spotted gar (*Lepisosteus oculatus*), an example of a bony fish that only went through two rounds of WGD, whereas two *Il4*/*Il13*-related genes (*Il4/13a* and *Il4/13b*) have been found in pufferfish (*Tetraodon nigroviridis*) and in zebrafish (*Danio rerio*) as a result of a third round of WGD that teleost fish went through [43]. Based on these findings, it is believed that a single *Il4*/*Il13* gene existed in ancestral gnathostomes, which has duplicated during WGD and/or tandem duplication during vertebrate evolution and thereafter evolved into the so-called type 2 cytokine locus including *Il4*, *Il5*, and *Il13* [40].

## The evolution of the IL-4 receptor system

IL-4 and IL-13 signal via heterodimeric IL-4Rs composed of three receptor subunits: IL-4Rα, the common gamma chain cytokine receptor (*γ*_c_), and IL-13Rα1 [8]. IL-4 signals via both the type 1 IL-4R composed of IL-4Rα and *γ*_c_ and the type 2 IL-4R composed of IL-4Rα and IL-13Rα1. IL-13 only signals via the type 2 IL-4R. In addition, IL-13 can interact with IL-13Rα2, which is thought to be a decoy receptor without signaling function.

IL-4R subunits are found in all jawed vertebrates (Fig. 1) [40]. All of the IL-4R and IL-13R genes belong to the class I cytokine receptors, which most likely originated from glycoprotein 130-like receptors present in invertebrates [44]. Although some class I cytokine receptor genes seem to have arisen from the two WGD, others have likely been created by tandem or *en bloc* gene duplication. The extra WGD the teleost lineage went through is possibly responsible for the unique teleost IL-4Rs and IL-13Rs. There is very sparse information available on cross-reactivity of the IL-4/IL-13–IL-4R system in different species [45].

IL-4Rα (also termed CD124), the shared receptor subunit of the type 1 and type 2 IL-4Rs, was identified in a large number of sequenced bird genomes [46]. Based on this, the gene was found to have an enhanced rate of nonsynonymous substitutions, and certain sites were classified as being under particularly high positive selection pressure. Interestingly, this might relate to a finding in the human *Il4ra* gene where some polymorphisms led to a higher susceptibility to asthma [47], and in mice where a single amino acid substitution in the *Il4ra* gene favored the development of asthma-like lung disease [48]. Again, the fishes undergoing a third round of WGD have two genes encoding for IL-4Rα (IL-4Rα1 and IL-4Rα2), and the *Il4ra* gene variants differ considerably between species. In zebrafish, alternative splicing results in a secreted IL-4Rα isoform found in liver, brain, and muscle tissue. Administration of zebrafish recombinant IL-4/13A showed in vivo effects including antibody production by B cells and CD40 expression, which is important for induction of type 2 immunity [49]. This serves as further proof that a well-developed adaptive immune system is already established in fishes.

*γ*_c_ (also known as CD132, encoded by the *Il2rg* gene) is a shared receptor subunit of the cytokines IL-2, IL-4, IL-7, IL-9, IL-15, and IL-21 [50]. *Il2rg* was found in both fishes and birds. Although the gene in birds is very similar to that of mammals, alternative splice variants exist [51]. *γ*_c_-expressing T cells in chicken have been shown to be important in fighting virus infections [52], thus demonstrating a genetic and functional similarity of *γ*_c_ between chickens and humans. In fish, *Il2rg* was initially identified in rainbow trout (*Oncorhynchus mykiss)* and was later found in zebrafish (*Danio rerio*), elephant shark (*Callorhinchus milii*), spotted gar (*Lepisosteus oculatus)*, and in several species of the *Tilapia* genus. Similar to *Il4ra*, some fishes have two paralogues of *Il2rg* [40]. Here, however, the mechanism giving rise to the duplication might not be solely due to a third round of WGD, as some species have the two genes on the same chromosome.

Less research has gone into investigating *Il13ra1* and *Il13ra2* (also termed CD213a1 and CD213a2, respectively), but also these two genes exist in all jawed vertebrates. From a functional perspective, both IL-13Rα1 and IL-13Rα2 are upregulated upon infectious stimuli in chicken [53, 54]. In trout, two paralogues of both *Il13ra1* and *Il13ra2* are present due to a third WGD; whereas the IL-13Rα2-related proteins (IL-13Rα2a/IL-13Rα2b) show 79% amino acid similarity, IL-13Rα1a and IL-13Rα1b have an amino acid identity of only 34% [55]. A distinctive expression pattern also applies to all of the subunits in trout: thus, IL-13Rα1b and IL-13Rα2b are primarily expressed in the ovaries, whereas IL-13Rα2a is expressed in spleen, head kidney, and mucosal tissue and IL-13Rα1a in scales, gills, and skin [56]. This suggests that the paralogues specialized to become tissue-specific.

Based on these findings, the expansion and development of the class I cytokine receptors and thereby the IL-4R and IL-13R subunits correlated with the appearance of adaptive immunity, which occurred together with a refinement of the innate immune system.

## Conclusion

Whereas neutrophil-like granular phagocytes were present in invertebrates throughout the bilaterian clade, we were unable to find data on IL-4, IL-13, IL-4Rα, IL-13Rα1, IL-13Rα2, and *γ*_c_ in invertebrates. Rather, IL-4, IL-13, and their receptors are found in vertebrates, thus coinciding with the phylogenetic development of a *bona fide* adaptive immune system. Notably, we did not find any evidence of type 2 cytokines in invertebrates, which could either indicate that these cytokines evolved later or could be due to a lack of data. The presence of eosinophilic and basophilic granular hemocytes in invertebrates could indicate a primal form of type 2 immunity, possibly harnessing factors that are upregulated during early phases of helminth infestations, such as arginase-1, chitinase-like protein 3, and resistin-like molecule α. However, how exactly these cells recognize and fight parasites will need to be further investigated. Future studies are necessary to determine whether IL-4R signaling in neutrophils always served a dual function in adaptive immunity and in curtailing neutrophil effector functions, or whether the neutrophil-specific function of IL-4R signaling evolved later. Moreover, the primary evolutionary source of IL-4/IL-13 production is still unknown and remains to be assessed in the future. Functional assays in phylogenetically older taxa, such as fishes, are needed to explore these questions.
